# Design and Optimization of a Pressure Sensor Based on Serpentine-Shaped Graphene Piezoresistors for Measuring Low Pressure

**DOI:** 10.3390/s22134937

**Published:** 2022-06-30

**Authors:** Xincheng Ren, Xianyun Liu, Xin Su, Xingfang Jiang

**Affiliations:** School of Microelectronics and Control Engineering, Changzhou University, Changzhou 213164, China; mexcren@163.com (X.R.); suxineerr@163.com (X.S.); xfjiang@cczu.edu.cn (X.J.)

**Keywords:** piezoresistive pressure sensor, graphene, finite element method, sensitivity, nonlinearity

## Abstract

This thesis describes a novel microelectromechanical system (MEMS) piezoresistive pressure sensor based on serpentine-shaped graphene piezoresistors paired with trapezoidal prisms under the diaphragm for measuring low pressure. The finite element method (FEM) is utilized to analyze the mechanical stress and membrane deflection to enhance the degree of stress concentration in this unique sensor. The functional relationship between mechanical performance and dimension variables is established after using the curve fitting approach to handle the stress and deflection. Additionally, the Taguchi optimization method is employed to identify the best dimensions for the proposed structure. Then, the suggested design is compared to the other three designs in terms of operating performance. It is revealed that the recommended sensor can significantly improve sensitivity while maintaining extremely low nonlinearity. In this study, three different types of serpentine-shaped graphene piezoresistors are also designed, and their sensing capability is compared to silicon. The simulation results indicate that the pressure sensor with Type 2 graphene piezoresistors has a maximum sensitivity of 24.50 mV/psi and ultra-low nonlinearity of 0.06% FSS in the pressure range of 0–3 psi.

## 1. Introduction

In recent years, piezoresistive pressure sensors based on microelectromechanical system (MEMS) have been employed in many industrial and commercial applications. Piezoresistive pressure sensors, manufactured utilizing the piezoresistive effect of semiconductor materials, have the benefits of excellent performance, a low price, small scale, and ease of preparation [1,2]. The diaphragm and piezoresistive elements are the essential parts of a piezoresistive pressure sensor, in which four piezoresistors are positioned in the high strain area of the diaphragm to form a full Wheatstone bridge. The diaphragm deforms to produce stress when external pressure is exerted, and the resulting change in the resistance of piezoresistors is transformed into an output voltage. Piezoresistive pressure sensors are now widely employed in aerospace [3], automotive [4], and biomedical [5] applications because of their numerous advantages.

However, the performance of MEMS piezoresistive pressure sensors can degrade due to the trade-off between sensitivity and nonlinearity. Two key factors, the geometric design of the sensor and the selection of piezoresistive material, are focused on alleviating the conflict between sensitivity and nonlinearity. Therefore, many structures have been suggested, debated, and extensively researched during the last few decades. For instance, Nambisan et al. compared the sensitivity and nonlinearity of a piezoresistive pressure sensor with a typical diaphragm to a piezoresistive pressure sensor with a bossed design to enhance the device’s performance. The study also discovered a linear trend in sensitivity and nonlinearity as the size of the introduced boss increased [6]. Tian et al. created a cross-beam-membrane (CBM) structure, with experimental findings indicating a sensitivity of 7.081 mV/kPa and a pretty low nonlinearity of 0.09% FSS, but the device was bulky [7,8]. Yu et al. presented a beam-membrane-quad-island (BMQI) structure with a comparatively high sensitivity of 17.795 μV/V/Pa but poor linearity and a reasonably massive device size [9,10]. Another pressure sensor with a peninsula structure situated on a diaphragm was proposed by Huang et al.; the sensitivity of this sensor was measured to be 18.4 mV/kPa, while the nonlinearity was 0.36% FSS and the device size was 3600 × 3600 μm^2^ [11]. Xu et al. suggested a novel diaphragm that combines a bossed design with a peninsula–island structure to decrease strain energy dissipation in locations other than the stress concentration. The sensitivity of this sensor was determined to be 0.066 mV/V/Pa, but its nonlinearity was 0.33% FSS, and its diaphragm size was 3500 × 3500 μm^2^ [12]. Meng et al. devised a piezoresistive pressure sensor with a beam-membrane-dual-island structure, with a sensitivity of 17.339 μV/V/Pa; however, the nonlinearity was relatively large, reaching 2.556% FSS, and the chip size was also huge at 7000 × 7000 μm^2^ [13]. A pressure sensor combining a four-grooved membrane with a rod beam was developed by Li et al., which has a sensitivity of up to 30.9 mV/V/psi and nonlinearity of 0.21% FSS [14]. Tran et al. designed a piezoresistive pressure sensor with a great sensitivity of 34.67 mV/kPa and nonlinearity of 0.23% FSS by integrating a four-petal membrane, four narrow beams, and a central boss (PMNBCB) [15]. Gao et al. suggested the development of a novel peninsula-shaped diaphragm piezoresistive differential pressure sensor. The sensitivity of this sensor was calculated to be 22.7 mV/kPa with a nonlinearity of 0.11% FSS [16]. However, these sensors that mix beams and islands all have poor linearity, limiting the application of pressure sensors in various fields.

For many years, monocrystalline silicon materials have been commonly used as piezoresistive components in pressure sensors. However, to further develop piezoresistive sensors with increased sensitivity, a broad detection range, and intense pressure resistance, several researchers have begun to focus on the novel two-dimensional (2D) material graphene [17]. Graphene has a Young’s modulus of up to 1 Tpa [18], exceptionally high stiffness, outstanding electrical conductivity [19], remarkable flexibility, and tensile strain of up to 18.7% [20,21]. Moreover, the resistivity of graphene changes linearly with strain [20,22]. As a result, graphene has become one of the most promising alternatives for piezoresistive materials. Using the contents of Singh et al.’s research on the size of piezoresistive pressure sensors [23], Nag et al. compared the sensing performance of graphene and polysilicon when they were employed as piezoresistive materials. The simulation results indicated that the sensitivity of the graphene pressure sensor, albeit 0.17 mV/psi higher than the polysilicon pressure sensor, was relatively low at 3.98 mV/psi [24]. Soon after, Nag et al. introduced rod beams placed beneath the film based on their previous study, which increased the sensitivity by 58%, but it was still low at 6.28 mV/psi [25]. Chun et al. suggested employing two separated single-layer graphenes on a flexible substrate to create a graphene pressure sensor for tactile sensing. For low-pressure measurements, the sensitivity was −0.24 kPa^−1^, and for high-pressure measurements, it was 0.039 kPa^−1^ [26]. Rinaldi et al. reported a multilayer graphene piezoresistive pressure sensor with a calculated sensitivity of 0.23 kPa^−1^ and the ability to detect compressive stresses as low as 10 kPa at an applied pressure of 70 kPa [27]. As observed, graphene is an excellent piezoresistive material for improving device performance.

**Figure 1:**
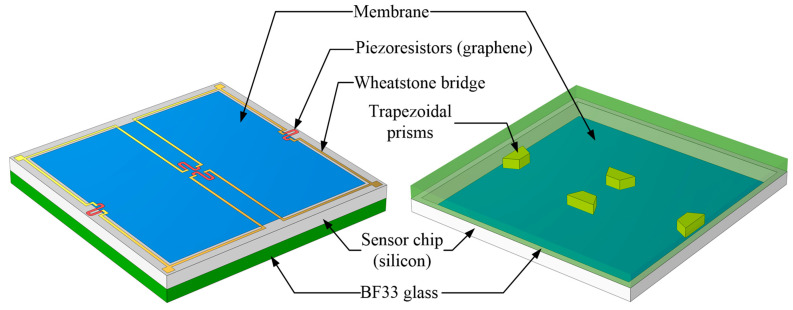
The 3D structure of the proposed pressure sensor.

This paper presents a unique structure of a graphene piezoresistive pressure sensor combining a flat diaphragm with trapezoidal prisms for detecting low pressure (see Figure 1). COMSOL Multiphysics software is used to conduct a finite element analysis (FEA) of the stress distribution and deflection for pressure sensors. The initial formulation and further optimization of the structural dimensions are then made using the curve fitting approach and Taguchi method. Moreover, the proposed structure is also compared to three other designs to demonstrate its superiority. Finally, three different serpentine-shaped graphene piezoresistors are explored to maximize sensor performance.

## 2. Fundamentals and Methodology

**Figure 2:**
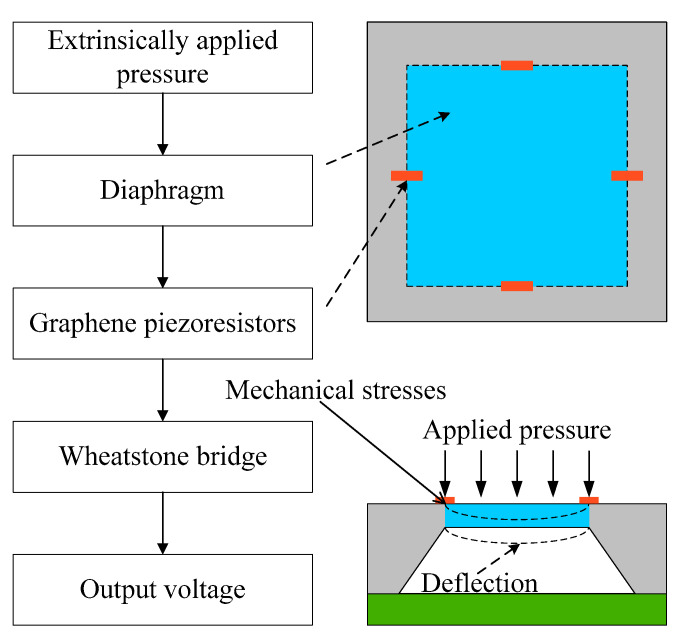
The primary working principle of a piezoresistive pressure sensor.

A piezoresistive pressure sensor comprises a thin plate and a complete Wheatstone bridge structure of four piezoresistors. Figure 2 describes the primary working principle of a piezoresistive pressure sensor, which is based on generated stress and deflection under extrinsically applied pressure. Once force is exerted on the top of the diaphragm, the piezoresistors are subjected to induced stress, and their resistance values fluctuate due to the piezoresistive effect, resulting in a change in the output voltage of the Wheatstone bridge structure. It is worth noting that the sensing element of a piezoresistive pressure sensor is critical in translating applied pressure into resistance and output voltage fluctuation.

**Figure 3:**
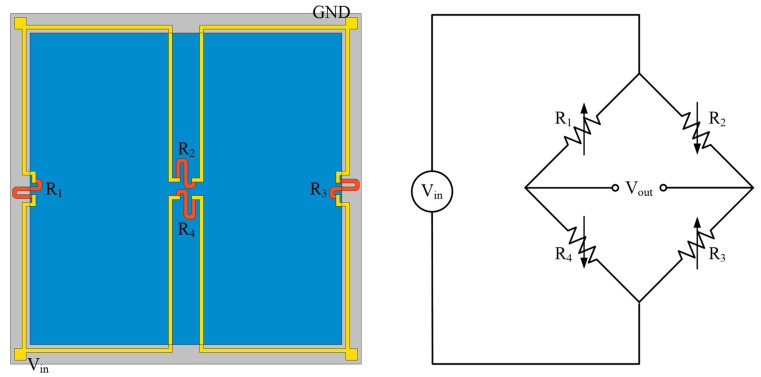
The positioning of four piezoresistors and Wheatstone bridge structure.

Since each piezoresistor is placed to cover the region of tensile or compressive stress, thus, two piezoresistors are put towards the central zone of the membrane, while the other two are positioned at the borders of the fixed diaphragm [23]. Figure 3 depicts the positioning of four piezoresistors coupled in a Wheatstone bridge construction, with *R*_1_ and *R*_3_ representing longitudinal piezoresistors and *R*_2_ and *R*_4_ representing transverse piezoresistors. In this work, the piezoresistors are manufactured by graphene and aligned along the (110)-direction on (100) n-type silicon to enhance the piezoresistive effect [28]. The deformation of the sensing element changes the potential distribution, which influences carrier mobility and, ultimately, the element resistance. The resistivity is determined by the sensor tensor and the 6 × 6 piezoresistive coefficient matrix. Due to the crystalline form of graphene, the piezoresistive coefficient matrix only has three non-zero independent components (*π*_11_, *π*_12_, and *π*_44_), as illustrated below

(1) $$\Pi =\left[\begin{array}{cccccc} \pi_{11} & \pi_{12} & \pi_{12} & 0 & 0 & 0\\ \pi_{12} & \pi_{11} & \pi_{12} & 0 & 0 & 0\\ \pi_{12} & \pi_{12} & \pi_{11} & 0 & 0 & 0\\ 0 & 0 & 0 & \pi_{44} & 0 & 0\\ 0 & 0 & 0 & 0 & \pi_{44} & 0\\ 0 & 0 & 0 & 0 & 0 & \pi_{44} \end{array}\right]$$

The piezoresistive coefficients of the graphene material utilized in this work are referenced from a similar investigation by Nag et al. [24]. For resistance fluctuations in a piezoresistor, the simplified formula can be represented by

(2) $$\frac{\Delta R}{R}=\pi_{l}\sigma_{l}+\pi_{t}\sigma_{t},$$

where ∆*R* represents the change in resistance after applying pressure, and *R* indicates the resistance value when no force is applied [29]. *π_l_* and *π_t_* denote the longitudinal and transverse piezoresistive coefficients, respectively. *σ_l_* and *σ_t_* are the longitudinal and transverse stresses, respectively. Depending on the orientation of the piezoresistors placed on the diaphragm, *π_l_* and *π_t_* can be displayed by Equation (3) [30].

(3) $$\left\{\begin{array}{l} \pi_{l,110}=\frac{\pi_{11}+\pi_{12}+\pi_{44}}{2}\\ \pi_{t,110}=\frac{\pi_{11}+\pi_{12}-\pi_{44}}{2} \end{array}\right.$$

Unlike silicon materials, the components π_11_ and π_12_ in the piezoresistive coefficient tensor of graphene are significant. As a result, the connection between structural stresses and relative resistance change for (110)-oriented piezoresistors in Figure 3 is provided by

(4) $$\left\{\begin{array}{l} \frac{\Delta R_{1}}{R_{1}}=\frac{\Delta R_{3}}{R_{3}}=\frac{1}{2}(\pi_{11}+\pi_{12})(\sigma_{i1}+\sigma_{j1})+\frac{\pi_{44}}{2}(\sigma_{i1}-\sigma_{j1})\\ \frac{\Delta R_{2}}{R_{2}}=\frac{\Delta R_{4}}{R_{4}}=\frac{1}{2}(\pi_{11}+\pi_{12})(\sigma_{i2}+\sigma_{j2})+\frac{\pi_{44}}{2}(\sigma_{i2}-\sigma_{j2}) \end{array}\right.,$$

where *σ_i_*_1_ means the average longitudinal stress on *R*_1_ and *R*_3_, *σ_j_*_1_ means the average transverse stress on *R*_1_ and *R*_3_; *σ_i_*_2_ denotes the average longitudinal stress on *R*_2_ and *R*_4_, and *σ_j_*_2_ represents the average transverse stress on *R*_2_ and *R*_4_ [31]. The relationship between the input and output voltages of the bridge can be described as

(5) $$V_{\mathrm{out}}=\frac{V_{\mathrm{in}}}{4}\left(\frac{\Delta R_{1}}{R_{1}}-\frac{\Delta R_{2}}{R_{2}}+\frac{\Delta R_{3}}{R_{3}}-\frac{\Delta R_{4}}{R_{4}}\right)$$

According to Equation (4), Equation (5) can be written as

(6) $$V_{\mathrm{out}}=\frac{V_{\mathrm{in}}}{4}\left[\left(\pi_{11}+\pi_{12})(\Delta \sigma_{\mathrm{ij}1}-\Delta \sigma_{\mathrm{ij}2})+\pi_{44}(\Delta \sigma_{\mathrm{ij}3}-\Delta \sigma_{\mathrm{ij}4}\right)\right],$$

where *σ_ij_*_1_ and *σ_ij_*_2_ signify the total of longitudinal and transverse stresses ($\Delta \sigma_{\mathrm{ij}1}=\sigma_{i1}+\sigma_{j1}$, $\Delta \sigma_{\mathrm{ij}2}=\sigma_{i2}+\sigma_{j2}$), respectively, and *σ_ij_*_3_ and *σ_ij_*_4_ represent the difference between longitudinal and transverse stresses ($\Delta \sigma_{\mathrm{ij}3}=\sigma_{i1}-\sigma_{j1}$, $\Delta \sigma_{\mathrm{ij}4}=\sigma_{i2}-\sigma_{j2}$) separately [15,32]. Furthermore, considering the piezoresistive coefficient of graphene is impacted by temperature and doping concentration, all of the designs in this work are created at ambient temperature (25 °C) with a graphene doping concentration of 1 × 10^−13^ cm^−3^ [33,34,35]. The sensor’s output voltage depends on the various stresses, as stated in Equation (6); therefore, ∆*σ_ij_* may be considered a crucial element in designing the sensor construction. Additionally, the two most essential indices of sensor performance are sensitivity (*S*) and nonlinearity (*NLr*), which are represented by

(7) $$S=\frac{V_{\mathrm{out}}(P_{\max})-V_{\mathrm{out}}(P_{\min})}{P_{\max}-P_{\min}}=\frac{\Delta V_{f}}{\Delta P_{f}},$$

(8) $$NL_{r}=\left[\frac{V_{\mathrm{out}}(P_{r})-\frac{V_{\mathrm{out}}(P_{\max})}{P_{\max}}\times (P_{r})}{V_{\mathrm{out}}(P_{\max})}\right]\times 100\%,$$

where *P_max_*, *P_min_*, and *P_r_* denote the maximum, minimum, and randomly tested loading pressures respectively; *V_out_*(*P_max_*), *V_out_*(*P_min_*), and *V_out_*(*P_r_*) represent the output voltages measured for *P_max_*, *P_min_*, and *P_r_*, respectively; and ∆*V_f_*, ∆*P_f_*, and *NLr* are the full-scale output voltage, full-scale applied pressure, and nonlinearity, respectively. It is also worth noting that nonlinearity may be positive or negative based on the tested points, and the maximum value determined is referred to as the nonlinearity of the sensor, which is commonly stated in % FSS.

## 3. Structure Design and Optimization

### 3.1. Structure Design

**Figure 4:**
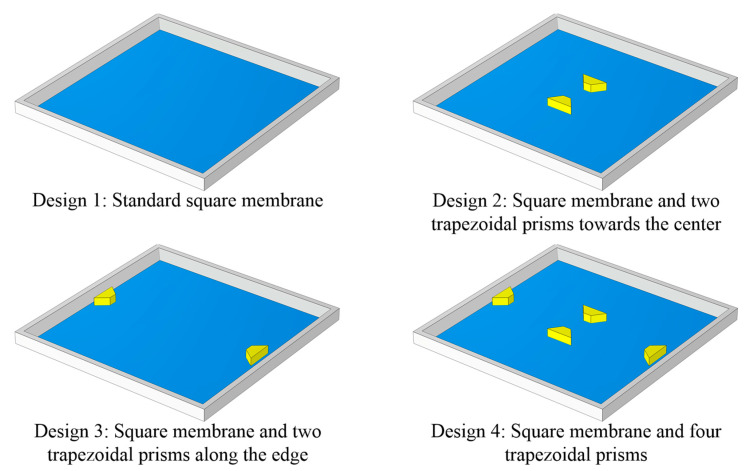
Four distinct designs of structures.

The device structure influences the magnitude of the sensitivity and nonlinearity in piezoresistive pressure sensors. In this study, a novel construction with trapezoidal prisms (two of the surfaces are isosceles trapezoids, and the other four are rectangles) is devised to increase the sensitivity of the pressure sensor while preserving excellent linearity. In addition, three other distinct design proposals are explored and contrasted with the new framework separately. Figure 4 illustrates these four specific designs, where Design 1 is a standard square diaphragm, Design 2 consists of a square diaphragm with two trapezoidal prisms towards the center of the diaphragm, Design 3 is a square diaphragm coupled with two trapezoidal prisms along the boundary of the diaphragm, and Design 4 is a hybrid of Designs 2 and 3. In addition, the primary parameters of these structures are designed to be consistent. Respectively, the length and thickness of these square membranes are 1000 μm and 12 μm. The dimensions of the trapezoidal prisms are 36 μm for the isosceles trapezoid upper base, 115 μm for the isosceles trapezoid lower base, 50 μm for the isosceles trapezoid height, and 35 μm for the thickness. Moreover, the trapezoidal prisms are added to the bottom of the membrane to generate a highly concentrated stress distribution zone, allowing the piezoresistors to endure more significant stresses and strains, thereby maximizing the sensitivity.

**Figure 5:**
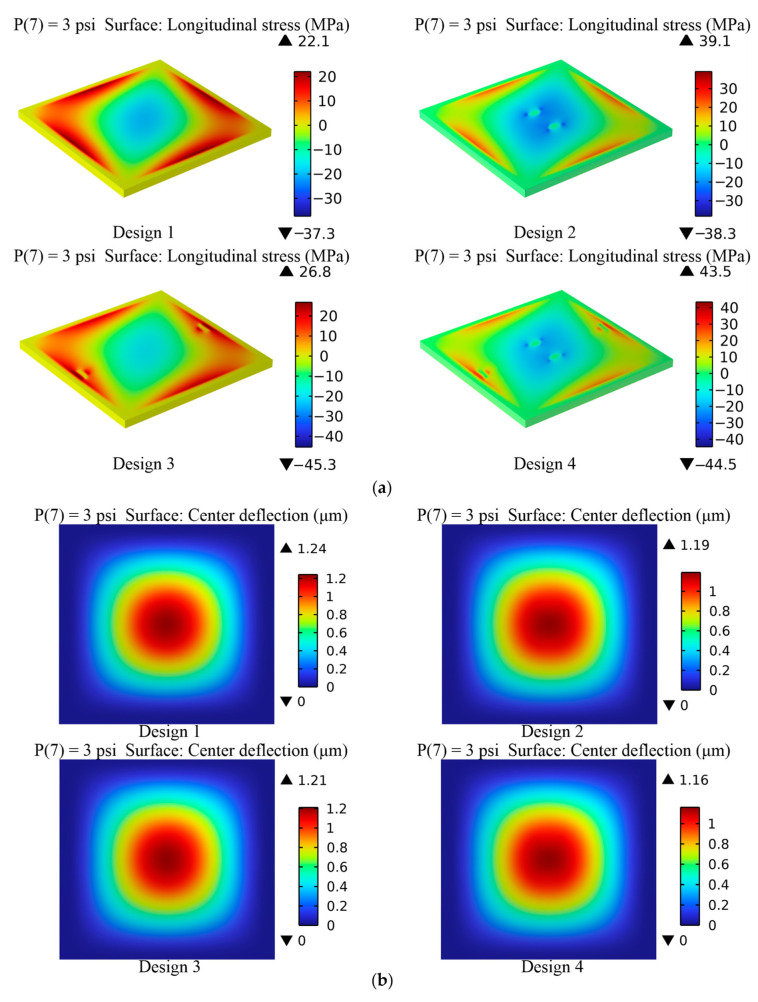
(**a**) The longitudinal stress of the four distinct constructions at 3 psi; and (**b**) the center deflection of the four distinct constructions at 3 psi.

Figure 5a,b illustrate the longitudinal stress and center deflection of the four different constructions when a 3 psi pressure is applied. The result indicates that Design 4 is subjected to significantly higher stresses than Designs 1, 2, and 3. Although the finding also implies that Designs 1, 2, and 3 all exhibit better deflection sensitivity than Design 4, the values of their maximum deflection are practically comparable. As a consequence of the analysis, it is determined that Design 4 is better suited for the micro pressure sensor. Furthermore, the deflection theory is classified into two types: small deflection theory and large deflection theory. The magnitude of the ratio between membrane deformation and thickness determines how they are distinguished. The large deflection theory holds when the deflection to thickness ratio is more than a particular value, resulting in a nonlinear relationship between deflection and pressure [36]. According to the big deflection curve, when the ratio of membrane deflection to membrane thickness exceeds 20%, the connection between the load pressure and deflection becomes nonlinear [37]. As a result, the small deflection theory operates when the most considerable deflection is less than one-fifth of the membrane thickness. The maximum deflection ratio to the membrane thickness (12 μm) for Design 4 is less than 0.2, indicating that it meets the criteria of the small deflection theory.

### 3.2. Mathematical Modeling

**Figure 6:**
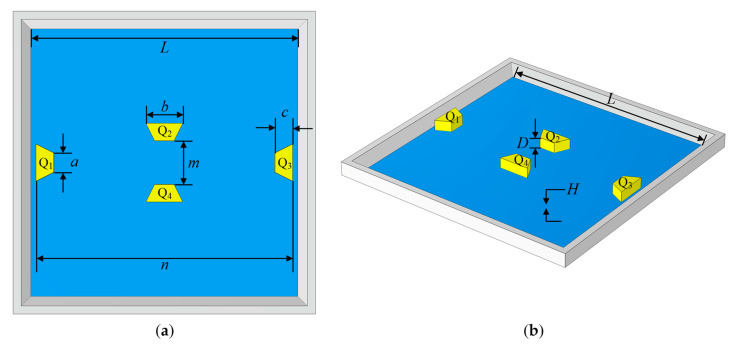
(**a**) Rear view and (**b**) isometric view of the proposed structure.

The theoretical formulas for the maximum stress and deflection of the intended new structure are derived using a mix of FEM calculations and the curve fitting approach in this work. The ultimate mechanical stress and deflection in a standard C-type construction are indicated in the expressions below

(9) $$\left\{\begin{array}{l} \sigma_{m}=0.308(1-\lambda^{2}){\left(\frac{L}{H}\right)}^{2}P=X_{1}L^{2}H^{-2}P\\ \omega_{m}=\frac{P}{47D_{FR}}{\left(\frac{L}{2}\right)}^{4}=\frac{12(1-\lambda^{2})P}{47EH^{3}}{\left(\frac{L}{2}\right)}^{4}=X_{2}L^{4}H^{-3}E^{-1}P \end{array}\right.,$$

where *λ* is the Poisson’s ratio, *L* is the membrane length, *H* is the membrane thickness, *P* is the applied pressure, *D_FR_* is the flexural rigidity, *E* is Young’s elastic modulus, and *X_1_* and *X*_2_ are coefficients to be determined [38,39]. The maximum stress and deflection of the membrane are power functions of each structural dimension variable, as illustrated in the calculation of the traditional C-type membrane in Equation (9) [40]. Thus, the functional form of the proposed new structure should be analogous to the C-type, as shown below

(10) $$\sigma_{\max}=X_{1}\cdot L^{y_{1}}\cdot H^{y_{2}}\cdot D^{y_{3}}\cdot a^{y_{4}}\cdot b^{y_{5}}\cdot c^{y_{6}}\cdot m^{y_{7}}\cdot N^{y_{8}}\cdot P^{y_{9}},$$

(11) $$\omega_{\max}=X_{2}\cdot L^{z_{1}}\cdot H^{z_{2}}\cdot D^{z_{3}}\cdot a^{z_{4}}\cdot b^{z_{5}}\cdot c^{z_{6}}\cdot m^{z_{7}}\cdot N^{z_{8}}\cdot E^{z_{9}}\cdot P^{z_{10}},$$

where *L*, *H*, *D*, *a*, *b*, and *c* are the novel structural dimensions illustrated in Figure 6; *m* and *n* denote the distances between trapezoidal prisms Q_2_ and Q_4_ and between the trapezoidal prisms Q_1_ and Q_3_, respectively; *X*_1_, *X*_2_, *y_r_* (*r* = 1, 2, …, 9), and *z_t_* (*t* = 1, 2, …, 10) are the curve fitting coefficients to be determined; *σ_max_* and *ω_max_* are the maximum von Mises stress and maximum deflection, respectively; *E* is Young’s elastic modulus, and *P* is the applied pressure.

Each variable must be evaluated to determine the above coefficients, while the other variables are treated as constants. For instance, when the effect of the membrane length *L* is investigated, the value of *L* is altered across a specific range, but other factors are considered to be constant. Thus, Equations (10) and (11) can be reduced to

(12) $$\sigma_{\max}(L)=X_{1l}\cdot L^{y_{1}},$$

(13) $$\omega_{\max}(L)=X_{2l}\cdot L^{z_{1}},$$

where *X*_1*l*_ and *X*_2*l*_ are undetermined coefficients for the variable *L*, and the factors of other variables have been taken into account. Due to the fluctuation in membrane length *L*, a series of *σ_max_* and *ω_max_* values will be provided by FEM numerical computation. Then, the coefficients *X*_1*l*_, *X*_2*l*_, *y*_1_, and *z*_1_ and fitting curves are acquired using MATLAB software. As a result, plugging the obtained coefficients into Equations (12) and (13) yields the following

(14) $$\sigma_{\max}(L)=1.588\times 10^{-4}\cdot L^{1.982}$$

(15) $$\omega_{\max}(L)=1.787\times 10^{-12}\cdot L^{4.01}$$

**Figure 7:**
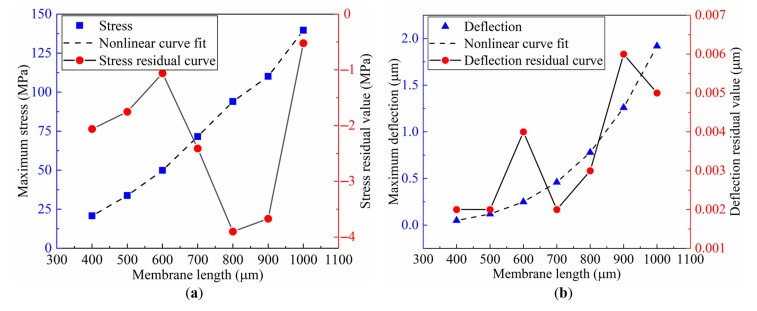
Membrane length variables versus mechanical performance variations: (**a**) maximum stress variation; and (**b**) maximum deflection variation.

Figure 7 depicts the influence of varied membrane lengths on stress and deflection and the residual curves of stress and deflection. When the length of the membrane expands, the maximum mechanical stress and maximum deflection increase as well. Furthermore, when the membrane length is 1000 μm, the residual value of stress is the smallest, and when the membrane length is 900 μm, the residual value of deflection is minimal. A membrane length of 1000 μm is chosen since the residual values of deflection corresponding to different film lengths do not change significantly. The coefficient of determination *R*^2^ and root mean squared error (*RMSE*) are offered to attain the optimum goodness of fit. According to the computation results of the software MATLAB, the values of $R_{\sigma_{\max}}^{2}$, $R_{\omega_{\max}}^{2}$, ${RMSE}_{\sigma_{\max}}$, and ${RMSE}_{\omega_{\max}}$ are 0.9961, 1, 2.929, and 0.0026, respectively. Thus, these findings reveal that the fitting curves and power functions match well with the simulated results.

The fitting curves and equations for other structural dimensions can also be determined using the same method. After acquiring all of the power function equations, these parameter values are substituted into Equations (10) and (11) to generate the equations utilized for the novel structure proposed in this study, as shown below

(16) $$\sigma_{\max}=X_{1}\frac{L^{1.982}a^{0.01683}c^{0.4623}m^{0.1096}n^{5.783}}{H^{2.009}D^{0.0278}b^{0.687}}P$$

(17) $$\omega_{\max}=X_{2}\frac{L^{4.01}m^{0.02226}}{H^{2.863}D^{0.007073}a^{0.008233}b^{0.09276}c^{0.0615}n^{0.1985}E}P$$

According to the magnitude of the residual values of the mechanical performance revealed by the fitting curves, the dimensional parameters of the proposed structure are chosen as *L* = 1000 μm, *a* = 26 μm, *b* = 116 μm, *c* = 48 μm, *H* = 12 μm, *D* = 35 μm, *m* = 120 μm, *n* = 870 μm, *E* = 160 GPa, and *P* = 3 psi. The values of *σ_max_* and *ω_max_* acquired from the simulation data are 85.20 MPa and 1.20 μm, respectively. Then, the values of the coefficients *X*_1_ and *X*_2_ can be calculated using the quantities mentioned above. As a result, the final expressions for maximum stress and deflection are derived below

(18) $$\sigma_{\max}=5.529\times 10^{-21}\frac{L^{1.982}a^{0.01683}c^{0.4623}m^{0.1096}n^{5.783}}{H^{2.009}D^{0.0278}b^{0.687}}P$$

(19) $$\omega_{\max}=1.792\times 10^{3}\frac{L^{4.01}m^{0.02226}}{H^{2.863}D^{0.007073}a^{0.008233}b^{0.09276}c^{0.0615}n^{0.1985}E}P$$

Equations (18) and (19) indicate that the membrane length *L*, membrane thickness *H*, and the distance *n* between the trapezoidal prisms Q_1_ and Q_3_ substantially influence stress and deflection. In other words, the variables *L*, *H*, and *n* all play a significant role in the sensitivity and linearity of the pressure sensor. The thickness *D* of the trapezoidal prism and the upper base *a* of its isosceles trapezoid have an essentially negligible influence on stress and deflection. Moreover, the lower base *b* and height *c* of the isosceles trapezoid in the trapezoidal prism and the distance *m* between the trapezoidal prisms Q_2_ and Q_4_ alter the stress amplitude but have nearly no effect on the membrane deflection. As a result of the above, it is feasible to improve the sensitivity of the pressure sensor while keeping a high level of linearity by setting the variables *m*, *n*, *b*, and *c* to the appropriate values.

### 3.3. Geometry Optimization

**Figure 8:**
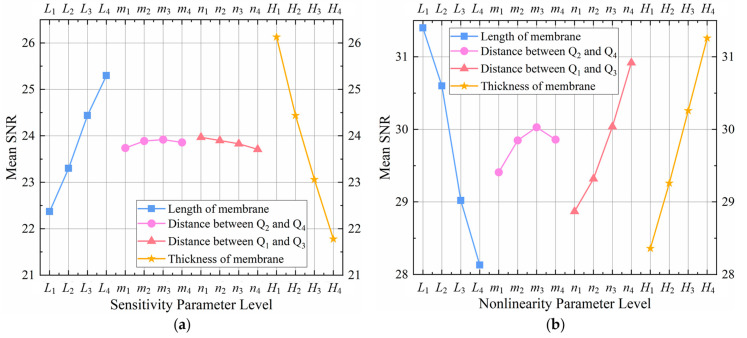
The mean SNR graphs for sensitivity and nonlinearity: (**a**) the larger-the-better SNR graph for sensitivity improvement; and (**b**) the smaller-the-better SNR graph for nonlinearity reduction.

In this section, the influence of each parameter on the sensitivity and linearity is further explored using the Taguchi optimization technique to discover the ideal geometric parameters of the suggested innovative construction. The Taguchi method employs a unique orthogonal array and signal-to-noise ratio (SNR) for the trial design, successfully lowering the design time without compromising quality [41,42]. In this work, since high sensitivity and low nonlinear error are required, the SNR calculation functions of “the-larger-the-better” and “the-smaller-the-better” proposed by Taguchi are adopted as follows

(20) $$\left\{\begin{array}{l} {\mathrm{SNR}}_{L}=-10\log\left[\frac{1}{n}\sum_{i=1}^{n}\frac{1}{y_{i}^{2}}\right]\\ {\mathrm{SNR}}_{S}=-10\log\left[\frac{1}{n}\sum_{i=1}^{n}y_{i}^{2}\right] \end{array}\right.,$$

where SNR*_L_* and SNR*_S_* denote the SNR for “the-larger-the-better” and “the-smaller-the-better”, respectively; *y_i_* is the output variable, and *n* is the number of observations. The four variables *L*, *m*, *n*, and *H* are first chosen and optimized, implying that the remaining variables are expected to remain constant when *L*, *m*, *n*, and *H* values vary. Table 1 shows the selected geometrical characteristics for the proposed structure, demonstrating four distinct levels at four variables (*L*, *m*, *n*, and *H*). Thus, the four-level coefficient settings of the Taguchi L_16_ (4^4^) standard orthogonal table in Minitab 19 software are utilized to analyze this study. After selecting the four variables mentioned earlier for optimization, Table 2 shows the simulation results and associated SNR for the sensitivity and nonlinearity of the tested pressure sensors. These findings are examined in Minitab software utilizing the SNR and the Pareto analysis of variance (ANOVA) approaches to improve the sensitivity and linearity simultaneously. In addition, Table 3 provides the response table of the average SNR for the sensitivity and nonlinearity for the L_16_ orthogonal table, and the mean SNR graphs for the sensitivity and nonlinearity are shown in Figure 8.

The best combination of the parameters for the four selected variables to maximize sensitivity can be determined by using the larger-the-better SNR method described in Figure 8a. The optimal dimensions are 1000 μm (*L*_4_) and 10 μm (*H*_1_) for the membrane length and thickness, respectively; 130 μm (*m*_3_) and 860 μm (*n*_1_) for the distances between trapezoidal prisms Q_2_ and Q_4_ and between trapezoidal prisms Q_1_ and Q_3_, respectively. Similarly, using the smaller-the-better SNR method depicted in Figure 8b, the ideal combination of the parameters for the four chosen variables that minimizes nonlinearity can be obtained. However, the perfect parameter of the variable *m* presented in Figure 8b is 120 μm (*m*_1_), which is the only one different from the parameters produced in Figure 8a. Table 3 and Figure 8 also reveal that the parameters of variables *L* and *H* have a significant impact on both sensitivity and nonlinearity, that the parameter of variable *n* has a more substantial effect on nonlinearity than sensitivity, and that the parameter of variable *m* has a minor impact on both sensitivity and nonlinearity. Thus, the parameter of the variable *m* is still set as 130 μm to enhance the performance of the pressure sensor.

**Figure 9:**
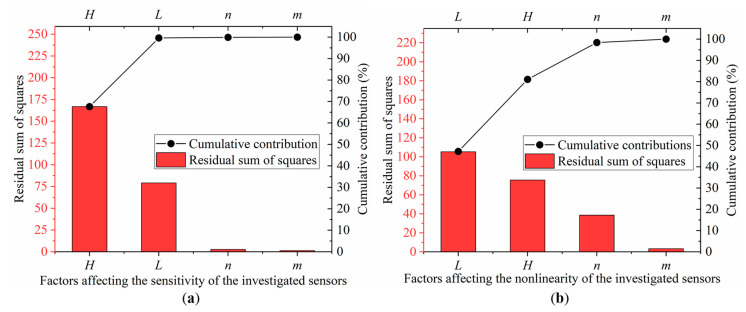
(**a**) The Pareto ANOVA diagram for sensitivity; and (**b**) the Pareto ANOVA diagram for nonlinearity.

Table 4 and Table 5 present the results of the Pareto ANOVA analysis for the sensitivity and nonlinearity of the investigated sensors separately. Pareto ANOVA is a simple and fast way to examine the outcomes of parameter designs based on the 80/20 rule. Furthermore, Figure 9 shows the Pareto ANOVA diagrams for the sensitivity and nonlinearity of the investigated sensors based on the data in Table 4 and Table 5. It is apparent that whether the Pareto ANOVA approach or the SNR method is utilized, they reach similar conclusions in terms of sensitivity improvement and nonlinearity reduction.

Similarly, the remaining variables can be optimized by utilizing the SNR and Pareto ANOVA approaches mentioned above. Finally, Table 6 demonstrates the ideal dimensions of the innovative structure presented in this thesis.

### 3.4. Simulation Comparison of Four Distinct Designs

**Figure 10:**
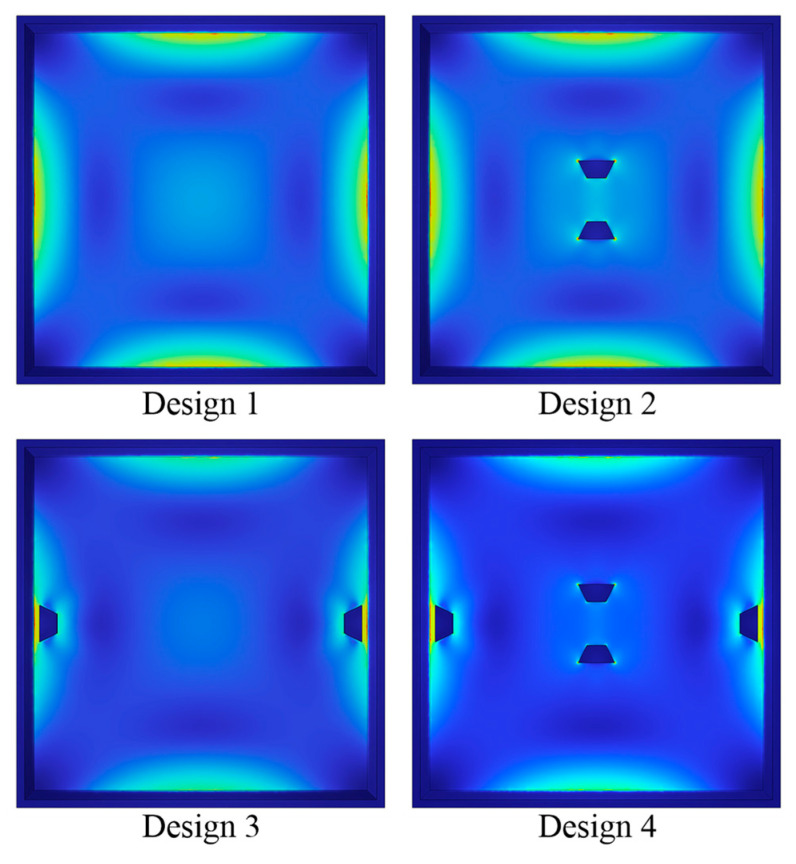
The four structures for simulation comparison.

**Figure 11:**
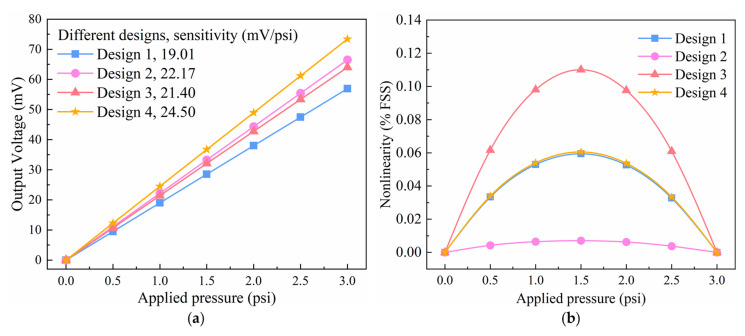
Comparison of the key performances for the four distinct designs: (**a**) the comparison of sensitivity and (**b**) the comparison of nonlinearity.

The four structures in Figure 10 are simulated, and the results are compared to highlight the superiority of the novel structure proposed in this research. The dimensions of these structures are reset to be consistent for scientific comparison based on the data in Table 6. Table 7 illustrates the center deflection and percentage of center deflection (deflection to membrane thickness) for each configuration under various pressure settings. Moreover, Figure 11 also demonstrates the primary performance of these pressure sensors at a 5 V input voltage and an applied pressure range of 0–3 psi.

According to a comprehensive analysis of the experimental data in Table 7 and Figure 11, the novel structure (Design 4) presented in this work has the optimum sensing performance in terms of sensitivity and linearity. The deflection percentages of Designs 1, 2, and 3 all surpass 20% when a loading pressure of 3 psi is applied; in other words, only Design 4 meets the condition of small deflection theory. Although the nonlinearities of Design 4 and Design 1 are virtually identical, the sensitivity of Design 4 is roughly 29% higher than Design 1. In addition, as compared to Design 3, the sensitivity of Design 4 is increased by about 14%, and the nonlinearity is decreased by approximately 45%. The nonlinearity of Design 4 is only 0.06% FSS. Since all designs have extremely modest nonlinear errors, Design 4 with the highest sensitivity is picked even though Design 2 shows the best linearity.

In general, modifying the structure and dimensions of the sensor may cause the sensitivity to change inversely to linearity. For instance, increasing the membrane thickness induces the sensitivity to drop while the linearity increases. In this study, trapezoidal prisms are added to the bottom of the membrane based on the location of the piezoresistors, all to subject the piezoresistors to more stress and decrease membrane deformation. Hence, the suggested device construction in this paper improves the sensitivity of the pressure sensor and maintains a very low nonlinearity. Finally, based on the findings above, the proposed structure is excellent and well suited for applying pressure sensors for micro pressure measurement.

## 4. Serpentine-Shaped Piezoresistor Design

**Figure 12:**
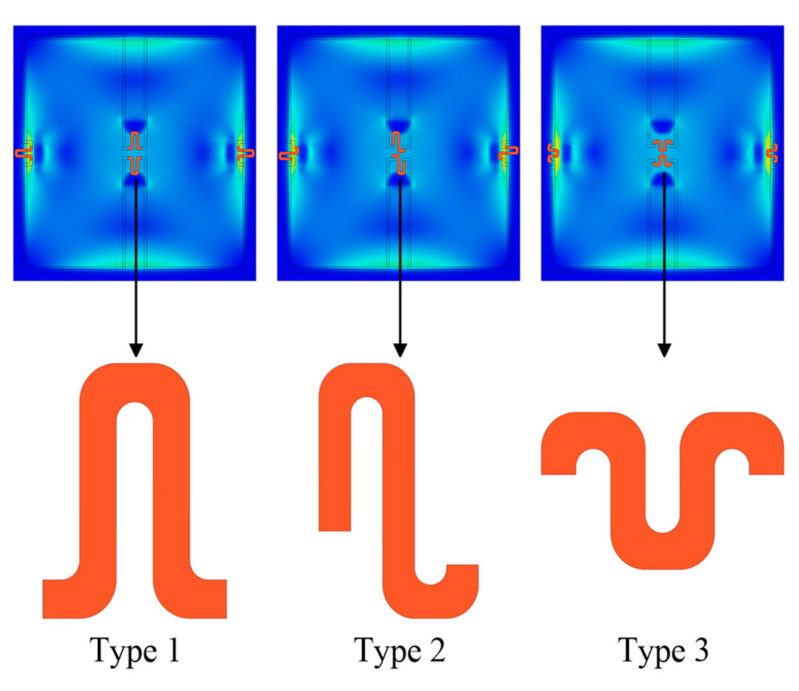
Three different serpentine-shaped piezoresistors.

This section presents three different serpentine-shaped piezoresistors, as shown in Figure 12. The performance of the three serpentine-shaped piezoresistors is compared, and it is worth noting that the length of these piezoresistors is the same. Therefore, the length and width of all three piezoresistors are set to 150 μm and 10 μm, respectively. The comparison of these three types of piezoresistors is performed in order to select a type that can withstand more stress and enable the sensor to possess excellent sensitivity while maintaining high linearity.

**Figure 13:**
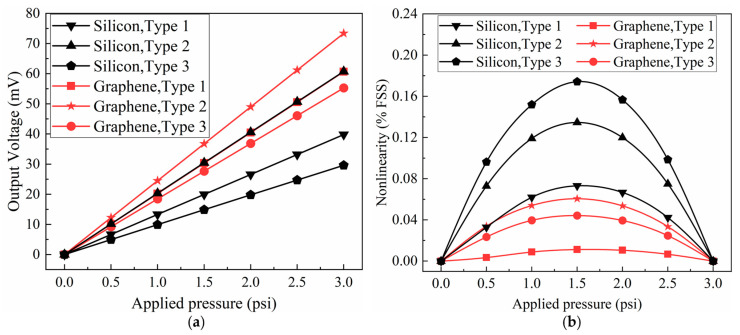
The impact of three different serpentine-shaped piezoresistors and two sensing materials on the significant sensor properties, respectively: (**a**) the effects on sensitivity and (**b**) nonlinearity.

Figure 13 depicts the influence of three distinct serpentine-shaped piezoresistors on sensitivity and nonlinearity and compares the piezoresistive properties between two sensing materials (graphene and silicon). The particular values of these sensitivities and nonlinearities are listed in Table 8. When graphene is used as the piezoresistive material, Type 2 has the highest sensitivity, almost 21% and 33% greater than Type 1 and Type 3, respectively, while Type 1 has the least nonlinearity. Similarly, when using silicon as the piezoresistive material, Type 2 still has the maximum sensitivity, approximately 53% higher than Type 1 and more than twice as high as Type 3, while Type 1 still has the lowest nonlinearity. Furthermore, regardless of whether graphene or silicon is used, all nonlinearities calculated by simulation are small, whereas sensitivity differences are substantial, making Type 2 more suitable for the proposed sensor. Figure 13 also illustrates that graphene outperforms silicon in sensitivity and nonlinearity, making it a better candidate for piezoresistive materials. Interestingly, the Type 2 piezoresistor made of silicon exhibits similar sensitivity to the Type 1 piezoresistor made of graphene, highlighting the superiority of Type 2.

To summarize, the Type 2 serpentine-shaped piezoresistor made of graphene is chosen to maximize the performance of the pressure sensor.

## 5. Manufacturing Process

**Figure 14:**
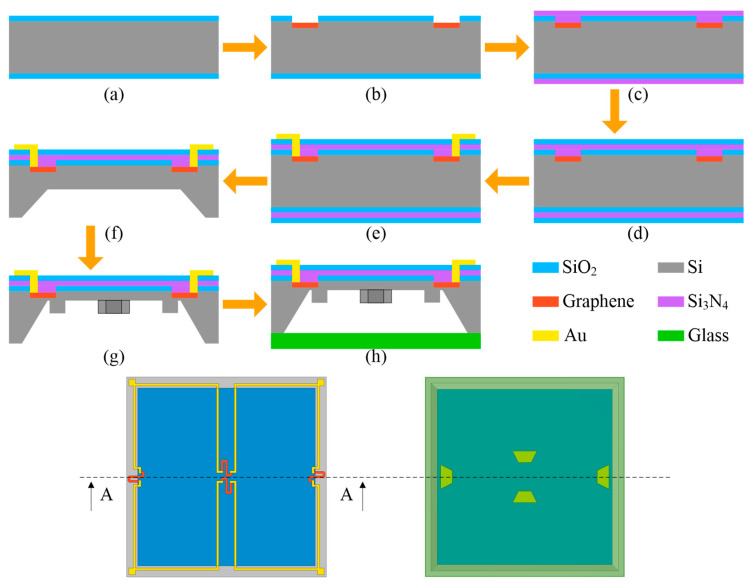
The primary manufacturing process of the suggested sensor chip.

A conventional n-type (100) silicon wafer with a thickness of 400 μm can be processed using bulk micromachining to create the sensor chip suggested in this work. Figure 14 illustrates the primary manufacturing procedure. Firstly, the selected silicon wafer is thermally oxidized to form thin layers of SiO_2_ on both of its surfaces (Figure 14a). Then, photolithography is used to process the placement region of the piezoresistors on the front side of the wafer to effectively transfer graphene. The piezoresistors are patterned by exposing the graphene layer with electron beams after applying the pattern mask (Figure 14b). Next, passivation layers of Si_3_N_4_ are generated using low-pressure chemical vapor deposition (LPCVD) to shield the piezoresistors (Figure 14c). Following that, SiO_2_ layers are deposited through the employment of the plasma-enhanced chemical vapor deposition (PECVD) technique to serve as electrical insulation (Figure 14d). Subsequently, reactive ion etching (RIE) and metallization processes are required to construct the connections between the piezoresistors so that Au can be effectively sputtered. As a result, the Wheatstone bridge circuit for the sensor chip is successfully fabricated (Figure 14e). Afterward, the backside of the wafer is handled with KOH etching to make an initial cavity (Figure 14f). To manufacture the proposed unique membrane structure with trapezoidal prisms, the backside of the wafer is once more processed utilizing the RIE technique (Figure 14g). Finally, BF33 glass is joined to the bottom of the sensor chip using an anodic bonding procedure, which increases the stability of the sensor by applying the glass as a stress buffer (Figure 14h).

## 6. Conclusions

This study creates a novel construction of a graphene piezoresistive pressure sensor with trapezoidal prisms and serpentine-shaped piezoresistors to improve the operating performance. The results of the mechanical stress and membrane deflection simulated by the FEM are evaluated to make this structure generate highly concentrated stress areas. The curve fitting method derives the equation between the mechanical characteristics and dimensional variables. Moreover, the Taguchi optimization approach is applied to obtain the optimal dimensions for the suggested structure. The proposed structure is then compared to three other device structures of various designs to find that it can effectively ease the conflict between sensitivity and linearity. This research also develops three distinct types of serpentine-shaped piezoresistors, evaluates the sensing properties of two piezoresistive materials, graphene and silicon, and concludes that the pressure sensor with Type 2 graphene piezoresistors has the most outstanding performance. The sensitivity of this graphene pressure sensor is up to 24.50 mV/psi, and the nonlinearity is just 0.06% FSS. Finally, the primary manufacturing procedures for the suggested sensor chip are provided. In summary, the proposed piezoresistive pressure sensor is an excellent alternative for low-pressure MEMS sensors.

## Figures and Tables

**Table 1:** Geometrical parameters and levels for the proposed structure.

Geometrical Parameters	Level 1	Level 2	Level 3	Level 4
*L*―Membrane length (μm)	850	900	950	1000
*m*―Distance between Q_2_ and Q_4_ (μm)	120	125	130	135
*n*―Distance between Q_1_ and Q_3_ (μm)	860	865	870	875
*H*―Membrane thickness (μm)	10	11	12	13

**Table 2:** Simulation results and associated SNR for sensitivity and nonlinearity of the tested pressure sensors.

Experimental Run	Factors				Simulated Parameters		Calculated SNR (dB)	
	*L*	*m*	*n*	*H*	Sensitivity (mV/psi)	Nonlinearity (%)	SNR for Sensitivity	SNR for Nonlinearity
1	1	1	1	1	17.0595	0.0372	24.6393	28.5891
2	1	2	2	2	14.2591	0.0297	23.0818	30.5449
3	1	3	3	3	12.0092	0.0239	21.5903	32.4320
4	1	4	4	4	10.1904	0.0199	20.1638	34.0229
5	2	1	2	3	13.2496	0.0310	22.4441	30.1728
6	2	2	1	4	11.6940	0.0273	21.3593	31.2767
7	2	3	4	1	18.9323	0.0294	25.5441	30.6331
8	2	4	3	2	15.5936	0.0305	23.8589	30.3140
9	3	1	3	4	12.9933	0.0300	22.2744	30.4576
10	3	2	4	3	15.0320	0.0295	23.5403	30.6036
11	3	3	1	2	18.2195	0.0409	25.2107	27.7655
12	3	4	2	1	21.7262	0.0434	26.7397	27.2502
13	4	1	4	2	19.0386	0.0379	25.5927	28.4272
14	4	2	3	1	23.9500	0.0449	27.5861	26.9551
15	4	3	2	4	14.6722	0.0343	23.3299	29.2941
16	4	4	1	3	17.1466	0.0405	24.6836	27.8509

**Table 3:** Response table of average SNR for sensitivity and nonlinearity for the L_16_ orthogonal table.

Factors	Mean SNR for Sensitivity (dB)					Mean SNR for Nonlinearity (dB)				
	Level 1	Level 2	Level 3	Level 4	Max–Min	Level 1	Level 2	Level 3	Level 4	Max–Min
*L*	22.37	23.30	24.44	25.30	2.93	31.40	30.60	29.02	28.13	3.27
*m*	23.74	23.89	23.92	23.86	0.18	29.41	29.85	30.03	29.86	0.62
*n*	23.97	23.90	23.83	23.71	0.26	28.87	29.32	30.04	30.92	2.05
*H*	26.13	24.44	23.06	21.78	4.35	28.36	29.26	30.26	31.26	2.91

**Table 4:** The Pareto ANOVA table for sensitivity.

Factor Levels	Factors Affecting the Sensitivity of the Investigated Sensors			
	*H*	*L*	*n*	*m*
1	104.51	89.48	95.89	94.95
2	97.74	93.21	95.60	95.57
3	92.26	97.77	95.31	95.68
4	87.13	101.19	94.84	95.45
Residual sum of squares (S)	166.72	78.98	0.60	0.31
Contribution ratio (%)	67.60	32.03	0.24	0.13
Cumulative contribution (%)	67.60	99.63	99.87	100.00

**Table 5:** The Pareto ANOVA table for nonlinearity.

Factor Levels	Factors Affecting the Nonlinearity of the Investigated Sensors			
	*L*	*H*	*n*	*m*
1	125.59	113.43	115.48	117.65
2	122.40	117.05	117.26	119.38
3	116.08	121.06	120.16	120.12
4	112.53	125.05	123.69	119.44
Residual sum of squares (S)	105.29	75.59	38.67	3.33
Contribution ratio (%)	47.24	33.92	17.35	1.49
Cumulative contribution (%)	47.24	81.16	98.51	100.00

**Table 6:** The ideal dimensions of the novel structure proposed in this study.

Variables	*L*	*H*	*m*	*n*	*D*	*a*	*b*	*c*
Dimension (μm)	1000	10	130	860	38	58	110	52

**Table 7:** Comparison of center deflection for the four structures under different applied pressures.

Pressure (psi)	Design 1	Design 2	Design 3	Design 4
0	0.00 μm	0.00 μm	0.00 μm	0.00 μm
0.5	0.37 μm	0.35 μm	0.35 μm	0.32 μm
1	0.73 μm	0.70 μm	0.70 μm	0.65 μm
1.5	1.10 μm	1.04 μm	1.06 μm	0.99 μm
2	1.46 μm	1.39 μm	1.41 μm	1.32 μm
2.5	1.83 μm	1.74 μm	1.76 μm	1.66 μm
3	2.19 μm	2.09 μm	2.11 μm	1.99 μm
Deflection percentage (%)	21.90	20.90	21.10	19.90

**Table 8:** Sensor characteristics for different types of graphene piezoresistors and silicon piezoresistors.

Materials	Sensitivity (mV/psi)			Nonlinearity (% FSS)		
	Type 1	Type 2	Type 3	Type 1	Type 2	Type 3
Graphene	20.22	24.50	18.44	0.01	0.06	0.04
Silicon	13.29	20.32	9.91	0.07	0.13	0.17
